# Characterization of TMAO productivity from carnitine challenge facilitates personalized nutrition and microbiome signatures discovery

**DOI:** 10.1186/s40168-020-00912-y

**Published:** 2020-11-19

**Authors:** Wei-Kai Wu, Suraphan Panyod, Po-Yu Liu, Chieh-Chang Chen, Hsien-Li Kao, Hsiao-Li Chuang, Ying-Hsien Chen, Hsin-Bai Zou, Han-Chun Kuo, Ching-Hua Kuo, Ben-Yang Liao, Tina H. T. Chiu, Ching-Hu Chung, Angela Yu-Chen Lin, Yi-Chia Lee, Sen-Lin Tang, Jin-Town Wang, Yu-Wei Wu, Cheng-Chih Hsu, Lee-Yan Sheen, Alexander N. Orekhov, Ming-Shiang Wu

**Affiliations:** 1 Department of Internal Medicine, National Taiwan University Hospital Bei-Hu Branch, Taipei, Taiwan; 2 Institute of Food Science and Technology, National Taiwan University, Taipei, Taiwan; 3 Department of Internal Medicine, College of Medicine, National Taiwan University, No. 7, Chung-Shan South Road, Taipei, 100 Taiwan; 4 Department of Internal Medicine, National Taiwan University Hospital, Taipei, Taiwan; 5 National Laboratory Animal Center, National Applied Research Laboratories, Taipei, Taiwan; 6 Department of Chemistry, National Taiwan University, Taipei, Taiwan; 7 The Metabolomics Core Laboratory, Center of Genomic Medicine, National Taiwan University, Taipei, Taiwan; 8 School of Pharmacy, College of Medicine, National Taiwan University, Taipei, Taiwan; 9 Institute of Population Health Sciences, National Health Research Institutes, Miaoli, Taiwan; 10 Department of Nutritional Science, Fu Jen Catholic University, Taipei, Taiwan; 11 Department of Medicine, Mackay Medical College, New Taipei City, Taiwan; 12 Graduate Institute of Environmental Engineering, National Taiwan University, Taipei, Taiwan; 13 Biodiversity Research Center, Academia Sinica, Taipei, Taiwan; 14 Graduate Institute of Microbiology, College of Medicine, National Taiwan University, Taipei, Taiwan; 15 Graduate Institute of Biomedical Informatics, College of Medical Science and Technology, Taipei Medical University, Taipei, 110 Taiwan; 16 Institute for Atherosclerosis Research, Skolkovo Innovative Center, Moscow, 121609 Russia; 17 Laboratory of Angiopathology, Institute of General Pathology and Pathophysiology, Moscow, 125315 Russia

**Keywords:** Gut microbiome, Trimethylamine N-oxide, Oral carnitine challenge test, Personalized nutrition, Cardiovascular disease, Machine learning, *Emergencia timonensis*, *Ihubacter massiliensis*

## Abstract

The capability of gut microbiota in degrading foods and drugs administered orally can result in diversified efficacies and toxicity interpersonally and cause significant impact on human health. Production of atherogenic trimethylamine N-oxide (TMAO) from carnitine is a gut microbiota-directed pathway and varies widely among individuals. Here, we demonstrated a personalized TMAO formation and carnitine bioavailability from carnitine supplements by differentiating individual TMAO productivities with a recently developed oral carnitine challenge test (OCCT). By exploring gut microbiome in subjects characterized by TMAO producer phenotypes, we identified 39 operational taxonomy units that were highly correlated to TMAO productivity, including *Emergencia timonensis*, which has been recently discovered to convert γ-butyrobetaine to TMA in vitro. A microbiome-based random forest classifier was therefore constructed to predict the TMAO producer phenotype (AUROC = 0.81) which was then validated with an external cohort (AUROC = 0.80). A novel bacterium called *Ihubacter massiliensis* was also discovered to be a key microbe for TMA/TMAO production by using an OCCT-based humanized gnotobiotic mice model. Simply combining the presence of *E*. *timonensis* and *I*. *massiliensis* could account for 43% of high TMAO producers with 97% specificity. Collectively, this human gut microbiota phenotype-directed approach offers potential for developing precision medicine and provides insights into translational research.

Video Abstract

Video Abstract

## Introduction

The gut microbiome stands at the intersection between diet and health [1]. Complexed interactions exist between foods, gut microbiota, and the host, which include the competition between human cells and gut microbes to absorb and utilize dietary ingredients from foods. Some beneficial nutrients or even drugs may be utilized by gut microbes to produce harmful metabolites for the human body [2–4]. For example, trimethylamine (TMA) is a microbiota-derived metabolite from dietary carnitine and choline, and its host-modified product, trimethylamine N-oxide (TMAO), was recently found to be highly correlated with cardiovascular disease (CVD) [5].

TMAO has been proven to aggravate CVD by promoting atherosclerosis and thrombotic risk and has also been reported to be correlated with other cardiometabolic diseases such as non-alcoholic fatty liver disease and chronic renal disease [6–8]. The pathological cutoff value of human plasma TMAO has not yet been concluded and it might benefit by referencing some current literatures [5, 6, 8–10]. In clinical aspects, Tang et al. reported that TMAO levels > 6.2 μM were significantly correlated to an increased risk of CVD events [5], whereas a recent dose-response meta-analysis demonstrated that the risk of all-cause mortality increased by 7.6% per 10 μM increment of plasma TMAO [7]. Mechanistically, the TMAO level for enhancing thrombosis potential was estimated to be at least 10–30 μM according to in vitro and animal studies [6, 10]. It implies chronic exposure to plasma TMAO > 10 μM in healthy subjects may have significant potential to cause unfavorable cardiometabolic risks. Nevertheless, TMAO formation from diet-gut microbiota-host interplay might be underestimated by observing fasting plasma TMAO levels because the kidney excretes TMAO efficiently to lower its blood levels [11].

To more accurately estimate TMAO productivity from carnitine metabolism in the human body, we have developed an oral carnitine challenge test (OCCT) to be applied as a gut microbiota functional test [11]. This test enables robust measurement of a participant’s TMAO production capacity from carnitine consumption and identification of relevant TMAO producer phenotypes in both omnivores and vegetarians. The OCCT may serve as a tool for directing a personalized carnitine intake because the biological fate of orally ingested carnitine varies interpersonally by gut microbiota and may cause paradoxical biological effects in patients [11]. For example, carnitine-enriched red meat has been suspected for decades as a cause of CVD, but research findings were inconsistent [12, 13]. Besides, carnitine supplementation was considered to benefit CVD patients by facilitating beta-oxidation of fatty acids but recent meta-analyses revealed conflicting results [14, 15]. These paradoxes may be attributed to the food-modifying effect of gut microbiota that has long been overlooked. Therefore, the OCCT may provide a personalized insight for carnitine consumption in health care by identifying TMAO productivity from host-microbe carnitine metabolism [11].

Characterization of TMAO productivity phenotypes by using OCCT may also help explore key human microbiome signatures of TMA synthesis from carnitine [11]. Although TMA-lyase has been characterized to be responsible for gut microbial choline metabolism [16–19], the key gut microorganism and the relevant enzyme involved in the conversion of carnitine to TMA remains unclear. Although a Rieske-type microbial *CntA*/*B* enzyme was proposed to convert carnitine to TMA [20], recent studies argued against its role in TMA synthesis from carnitine in the human gut [11, 21]. In fact, the *CntA*/*B* is an oxygen-dependent enzyme, and its function may be limited in the anaerobic gut environment. Instead, an anaerobic mechanism, carnitine ➔ γ-butyrobetaine (γBB) ➔ TMA, was proposed as a more convincing pathway for TMA generation from carnitine [21]. This multistep pathway was recently found to be mediated cooperatively by several members of human commensals, and a novel obligate anaerobe, *Emergencia timonensis*, was noted to convert γBB to TMA [21]. Therefore, the *E*. *timonensis* might explain a portion of TMAO production from carnitine consumption in human but still required to be validated with a larger cohort. Moreover, there might be other microbial contributors for converting γBB to TMA which also requires to be elucidated [2].

In the present study, we used OCCT as a TMAO-producer phenotype classifier to estimate TMAO formation from the intervention of carnitine supplementation. More importantly, given the efficacy of OCCT in identifying human TMAO productivity, we explored the gut microbial community contributing to TMA/TMAO production from carnitine metabolism in the human body.

## Results

### OCCT estimates TMAO production from carnitine supplementation

**Fig. 1:**
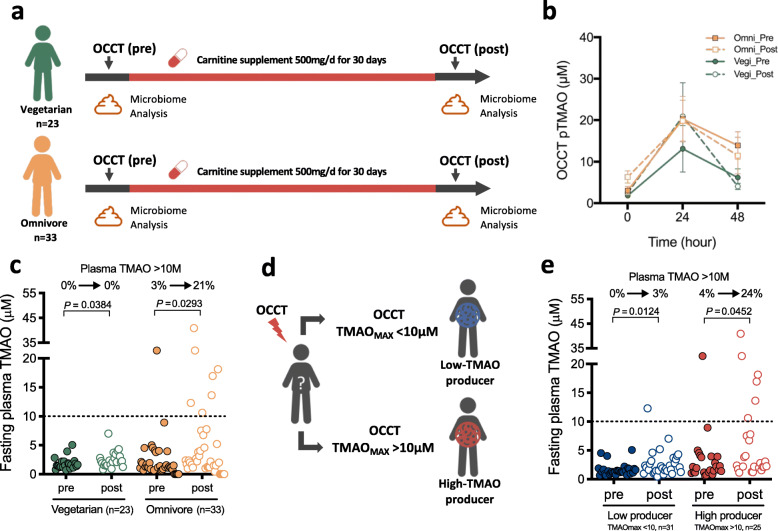
The oral carnitine challenge test (OCCT) can be used to estimate potentially harmful plasma TMAO levels from carnitine intake. **a** Schematic of the OCCT procedure and study protocol. **b** The OCCT curves for vegetarians and omnivores pre- and post-carnitine supplementation. **c** Fasting plasma TMAO levels in vegetarians and omnivores pre- and post-carnitine supplementation. **d** Classification of low- and high-TMAO producers based on a cut-off value of 10 μM for TMAO_MAX_ in the OCCT. **e** Fasting plasma TMAO levels in low- and high-TMAO producers pre- and post-carnitine supplementation. Data shown here were analyzed by paired and unpaired nonparametric test accordingly; bars represent the mean ± S.E.M for the indicated groups

The human gut commensals utilize the ingested carnitine interpersonally and cause variations in plasma TMAO levels. Here, we used our recently established OCCT to measure TMAO productivity in 56 individuals who received carnitine supplementation for 1 month (Fig. 1a). The OCCT was performed before and after carnitine supplementation (Fig. 1b). The plasma TMAO were significantly elevated in both omnivores and vegetarians, whereas plasma carnitine exhibited no obvious change (Fig. 1c and Table 1). After the 1-month carnitine supplementation intervention, the proportion of omnivorous subjects with potentially harmful plasma TMAO > 10 μM increased from 3% (1/33) to 21% (7/33), whereas plasma TMAO remained at < 10 μM in vegetarians (Fig. 1c). Urine TMAO level was also significantly elevated in omnivores rather than in vegetarians (Table 1).

**Table 1:** Characteristics of study participants before and after 1 month of carnitine intake

	Vegetarian pre-carnitine (*n* = 23)	Vegetarian post-carnitine (*n* = 23)	*p* value	Omnivore pre-carnitine (*n* = 33)	Omnivore post-carnitine (*n* = 33)	*p* value
Female (%)	61	–	–	73	–	–
Age (years)	34.13 ± 1.70	–	–	29.79 ± 1.28	–	–
BMI (kg m^-2^)	22.40± 0.55	22.51 ± 0.58	0.124	21.78 ± 0.58	21.76 ± 0.57	0.712
Plasma						
Glucose-AC (mg/dL)	69.39 ± 2.21	75.65 ± 3.23	0.071	75.21 ± 1.96	79.30 ± 1.23	0.048*
AST (U/L)	11.30 ± 1.04	13.04 ± 1.40	0.167	15.39 ± 1.22	14.06 ± 1.41	0.367
ALT (U/L)	7.22 ± 0.94	8.26 ± 1.21	0.335	10.97 ± 1.41	9.03 ± 0.91	0.064
BUN (mg/dL)	9.60 ± 0.65	15.64 ± 4.60	0.200	11.62 ± 0.51	12.02 ± 0.53	0.422
Creatinine (mg/dL)	0.55 ± 0.03	0.51 ± 0.03	0.175	0.59 ± 0.02	0.61 ± 0.02	0.354
T-Cholesterol (mg/dL)	140.52 ± 4.27	144.30 ± 5.19	0.436	174.76 ± 5.39	178.94 ± 4.84	0.297
Triglyceride (mg/dL)	89.13 ± 11.31	97.96 ± 11.88	0.183	86.18 ± 10.15	80.58 ± 6.70	0.353
LDL-C (mg/dL)	75.13 ± 4.25	80.83 ± 4.55	0.100	97.42 ± 4.78	102.33 ± 4.84	0.154
CRP C (mg/dL)	0.06 ± 0.02	0.09 ± 0.03	0.307	0.19 ± 0.09	0.09 ± 0.02	0.292
TMAO (μM)	1.82± 0.21	2.49 ± 0.28	0.038*	3.05 ± 0.98	6.31 ± 1.47	0.029*
Carnitine (μM)	32.23 ± 1.25	34.55 ± 1.48	0.097	34.16 ± 1.20	32.86 ± 1.44	0.193
Urine						
TMAO (nmol/mmol Cr)	33.98 ± 9.49	46.70 ± 6.19	0.160	53.62 ± 15.21	110.63 ± 26.21	0.015*
Carnitine (nmol/mmol Cr)	2.52 ± 0.63	8.43 ± 2.11	0.012*	15.76 ± 6.53	18.27 ± 5.92	0.777

* *P* values were obtained for the comparison of vegetarian and omnivore participants before and after carnitine supplementation by using a paired *t* test. Values are expressed as the mean ± S.E.M. *AST* aspartate aminotransferase, *ALT* alanine aminotransferase, *BUN* blood urea nitrogen, *LDL*-*C* low-density lipoprotein cholesterol, *CRP C* C-reactive protein, *TMAO* trimethylamine N-oxide

We then regrouped the subjects to estimate TMAO formation from carnitine supplements based on the pre-supplement OCCT results. We selected 10 μM as the cutoff value mentioned previously [5, 6, 8–10] for the maximal plasma level in the OCCT (OCCT TMAOMAX) for defining TMAO productivity phenotypes (Fig. 1d). Accordingly, 45% of healthy volunteers were categorized as high-TMAO producers, whereas 55% were grouped as low-TMAO producers. Approximately 26% of vegetarians exhibited remarkable TMAO productivity and were grouped as high-TMAO producers. Both low- and high-TMAO producers have a significant increase in fasting plasma TMAO levels after carnitine supplementation (Fig. 1e); however, the high-TMAO producers exhibited a greater increase as compared with low producers (mean 3.69 μM to 7.27 μM vs. 1.62 μM to 2.71 μM) (Fig. 1e). Notably, the percentage of fasting plasma TMAO > 10 μM increased from 4 to 24% in high-TMAO producers, whereas only 1 in 31 low-TMAO producers (3%) presented plasma TMAO > 10 μM after carnitine supplementation. The results indicated that a potentially harmful plasma TMAO level from carnitine supplementation could be estimated using OCCT-defined TMAO producer phenotypes.

### High-TMAO producers have reduced oral carnitine bioavailability

The TMAO productivity of gut microbiota may also present a personalized bioavailability of carnitine supplementation. To estimate the bioavailability of ingested carnitine, we first compared the fasting plasma carnitine before and after carnitine supplementation; no difference was observed in the plasma carnitine levels, whereas a significant increase was observed in the urine carnitine levels (Figure S1a left panel). By contrast, both plasma and urine TMAO levels were significantly increased after carnitine supplementation (Figure S1a right panel). We then investigated the underlying reason for the difference between carnitine and TMAO homeostasis. In our study, data revealed that the plasma and urine TMAO levels were highly correlated (Figure S1b), whereas the correlation between plasma and urine carnitine levels was modest and nonlinear (Figure S1c). The scatter plot reveals that the plasma carnitine levels were maintained at 20–60 μM at various renal excretory levels. These findings indicate that the homeostasis of carnitine and TMAO regulated by the kidney are considerably different.

We then compared the renal efficiency of carnitine and TMAO excretion by using fractional excretion of carnitine (FeCarnitine) and fractional excretion of TMAO (FeTMAO) referenced with a clinical formula, FeNa [22]. The results revealed a median FeCarnitine of 0.36% and a median FeTMAO of 93.85% (Figure S1d), which suggested that the kidneys strongly conserve carnitine in and eliminate TMAO from the human body (Figure S1e). Moreover, FeCarnitine was upregulated after a 1-month carnitine supplementation intervention, whereas FeTMAO levels did not change significantly (Figure S1d). Therefore, our data suggested that urine may be more suitable for estimating the bioavailability of ingested carnitine than plasma in this study design.

**Fig. 2:**
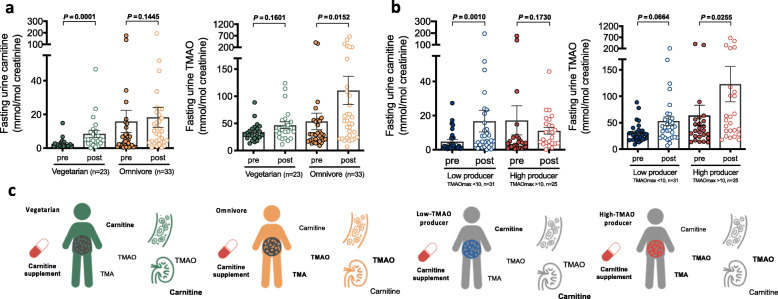
Orally ingested carnitine bioavailability is associated with gut microbiota TMAO productivity. **a** The fasting urine carnitine was significantly increased in the vegetarian group after carnitine supplementation, but not in the omnivorous group. The fasting urine TMAO was significantly increased in the omnivorous group after carnitine supplementation, but not in the vegetarian group. **b** The fasting urine carnitine was significantly increased in the low-TMAO producer group after carnitine supplementation, but not in the high-TMAO producer group. The fasting urine TMAO level was significantly increased in the high-TMAO producer group after carnitine supplementation, but not in the low-TMAO producer group. For **a** and **b**, data were analyzed by paired nonparametric test. Bars represent the mean ± s.e.m for the indicated groups. **c** Schematic diagram showing carnitine bioavailability which might be influenced by the ability of gut microbiota in converting carnitine into TMA

We thus found that urine carnitine increased significantly only in the vegetarian group (2.52 vs 8.42 mmol/mol creatine, *p* = 0.001), whereas urine TMAO was only remarkably elevated in the omnivorous group (53.62 vs. 110.63 mmol/mol creatine, *p* = 0.015) (Fig. 2a). Moreover, a remarkable increase in urine carnitine was observed low-TMAO producers (4.79 vs. 16.74 mmol/mol creatine, *p* = 0.001), but a mild nonsignificant decrease in urine carnitine was observed in high-TMAO producers (17.18 vs. 11.12 mmol/mol creatine, *p* = 0.173) (Fig. 2b). The increase in urine TMAO was also significant in high-TMAO producers rather than in low-TMAO producers. Taken together, our data suggested an obvious reduction in carnitine bioavailability in individuals with higher TMAO productivity, probably because a considerable portion of ingested carnitine was consumed and converted to TMA by the gut microbiota (Fig. 2c).

### TMAO productivity can be enhanced by carnitine supplementation

Studies have demonstrated that omnivores produce more TMAO from carnitine than do vegetarians [5, 11, 21]. This finding is probably because an omnivore’s gut microbiota is exposed to a relatively carnitine-rich environment and some carnitine-utilizing bacteria may be enriched. We then investigated whether carnitine supplementation to a regular diet would increase the TMAO productivity from carnitine utilization.

**Fig. 3:**
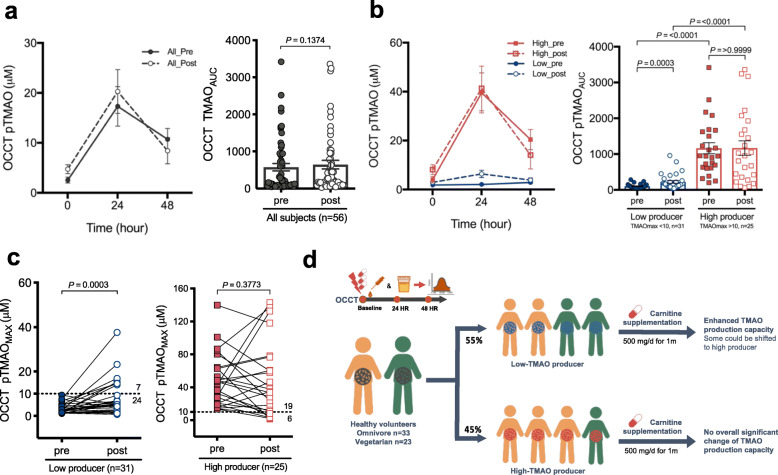
Gut microbiota TMAO productivity in low producer can be enhanced by carnitine supplementation. **a** For all the 56 subjects, the TMAO productivity showed nonsignificant change by carnitine supplementation. **b** For the subgroup of low-TMAO producer, TMAO productivity was significantly increased by carnitine supplementation, when it showed nonsignificant change in the subgroup of high-TMAO producer. For A and B, time reflects hours after oral challenge of carnitine. Bars represent the mean ± S.E.M for the indicated groups. **c** TMAO productivity represented by plasma TMAO_MAX_ of OCCT in the low-TMAO group was also increased significantly by carnitine supplementation, whereas it showed nonsignificant change in the high-TMAO producer group. **d** Schematic diagram briefly showing results of carnitine supplement intervention in healthy omnivores and vegetarians with each individual’s TMAO productivity measured by OCCT before and after the intervention. Generally, 55% subjects were grouped as low-TMAO producer phenotype while 45% subjects were grouped as high-TMAO producer. The TMAO production capacity in low-TMAO producer was significantly enhanced by carnitine supplementation with some shifted to high-TMAO producer phenotype. For the high-TMAO producer group, no overall significant change of TMAO productivity was found by the intervention

TMAO productivity measured using the OCCT is expressed as the area under the OCCT curve (OCCT TMAOAUC) or the maximum TMAO value of OCCT (OCCT TMAO_MAX_). Overall, no significant change was observed in TMAO productivity after the carnitine intervention (TMAO_AUC_ 574.70 vs. 644.88, *p* = 0.137) (Fig. 3a). Notably, TMAO productivity in low-TMAO producers was significantly elevated (TMAO_AUC_ 100.01 vs. 222.87, *p* = 0.0003), whereas that in high-TMAO producers exhibited no significant change (TMAO_AUC_ 1163.21 vs. 1168.17, *p* > 0.9999) (Fig. 3b). These findings were further confirmed by TMAO levels in urine samples (Figure S2a-b). We then hypothesized that different dietary habits may modify the effects of carnitine supplementation on TMAO productivity, and still found that TMAO productivity in low producers increased significantly regardless of their dietary patterns (Figure S2c-d); however, the productivity was still considerably lower than that of high-TMAO producers.

Furthermore, we assessed the proportion of individuals with initially low TMAO productivity who would potentially incur a harmful TMAO productivity upon additional carnitine consumption. We found seven low-TMAO producers (three omnivores and four vegetarians) were transformed to high producers after the 1-month carnitine supplementation intervention (Fig. 3c). The results suggested that supplemental carnitine intake to an individual’s regular diet may significantly increase TMAO productivity in individuals with low TMAO productivity, regardless of omnivorous or vegetarian dietary habits (Fig. 3d).

### TMAO producer phenotypes are distinguished by their gut microbiome profiles

**Fig. 4:**
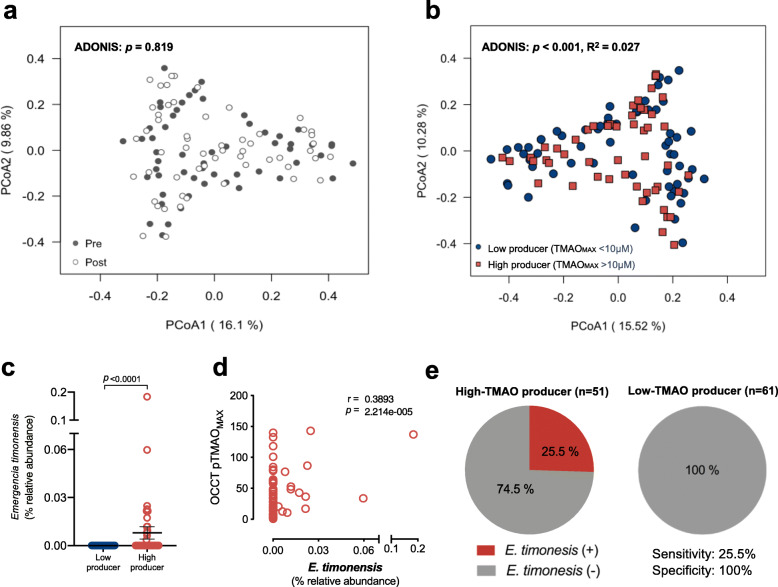
Distinct microbiome signatures were found between low- and high-TMAO producing status. **a** For all the 56 healthy subjects, the 16S rRNA gut microbial profiles did not show a significant change between pre- and post-carnitine supplementation. **b** When the samples were regrouped based on the status of TMAO productivity (i.e., TMAO_MAX_ < 10 μM vs. TMAO_MAX_ > 10 μM), the 16S rRNA gut microbial profiles exhibited a significant difference between low- and high-TMAO producers (*p* < 0.001, *R*^2^ 0.027). **c** The relative abundance of *E*. *timonensis* was significantly enriched in high-TMAO producers than low-TMAO producers. Data were analyzed by paired Wilcoxon test; bars represent the mean ± S.E.M for the indicated groups. **d** The relative abundance of *E. timonensis* was significantly correlated to the TMAO productivity. **e** The *E*. *timonensis* was present in 13 of 51 samples of high-TMAO producing status while it was absent in all 61 samples of low-TMAO producing status, contributing to a sensitivity of 25.5% with 100% specificity for detecting high-TMAO producers

Because the participants’ TMAO productivity was partially increased by carnitine supplementation, the gut microbial community may also have been altered. We then compared 16S rRNA gut microbial profiles of fecal samples collected before and after the carnitine intervention. Nevertheless, no significant alteration in microbial profiles was noted by carnitine supplementation, even for subgroup analysis of vegetarians and low-TMAO producers (Fig. 4a and Figure S3a-b). We then regrouped the 112 fecal samples collected from the 56 subjects before and after carnitine supplementation, based on its corresponding status of TMAO producing capacity: 61 samples were grouped as low-TMAO producer (TMAO_MAX_ < 10 μM) and 51 samples were grouped as high-TMAO producer (TMAO_MAX_ > 10 μM) according to the OCCT results. Consequently, a significant difference of 16S rRNA microbial profiles was noted between low- and high-TMAO producing status (*p* = 0.001, *R*^2^ = 0.027) despite a small effect size (Fig. 4b). We have also conducted another group comparison by separating samples between fasting TMAO < 6.2 μM versus fasting TMAO > 6.2 μM, and did not find significant difference of the microbial profiles (Figure S4a). It is probably because a significant portion of high TMAO producers (TMAO_MAX_ > 10 μM) were not detected by a simpler screening of fasting TMAO levels (Figure S4b-c).

We then compared all 1637 OTUs between the low- and high-TMAO producer groups and found 101 OTUs were significantly enriched in the high-TMAO producer group (FDR < 0.01) (Figure S5). Notably, the *E*. *timonensis* was included which is a novel bacterium recently found to be able to convert γBB into TMA_,_ while the microbiota-dependent γBB ➔ TMA pathway was believed to be pivotal for TMAO formation from carnitine intake [21].

In addition, the relative abundance of *E*. *timonensis* was significantly higher in the OCCT TMAOMAX > 10 μM group (0 vs. 0.008%, *p* < 0.0001) (Fig. 4c), but not in the group of fasting TMAO > 6.2 μM (Figure S4d). It was also significantly correlated to TMAO productivity from carnitine metabolism (Fig. 4d). Surprisingly, all identified *E*. *timonensis* were found exclusively in fecal samples grouped as high-TMAO producer (13/51, 25.5%, 8 from omnivores, 5 from vegetarians), whereas no *E*. *timonensis* bacterium was detected in the fecal samples of the low-TMAO producer group (0/61) (Fig. 4e). These findings provide further evidences for *E*. *timonensis* as an important microbial contributor for TMAO generation from carnitine intake. However, the presence of *E*. *timonensis* seems to only explain 25.5% of high-TMAO production in subjects and was noted in 11.6% (13/112) of all fecal samples. So, there could be other unrecognized bacteria responsible for this novel and important pathway.

### Gut microbiome features were identified to establish a TMAO producer prediction model

**Fig. 5:**
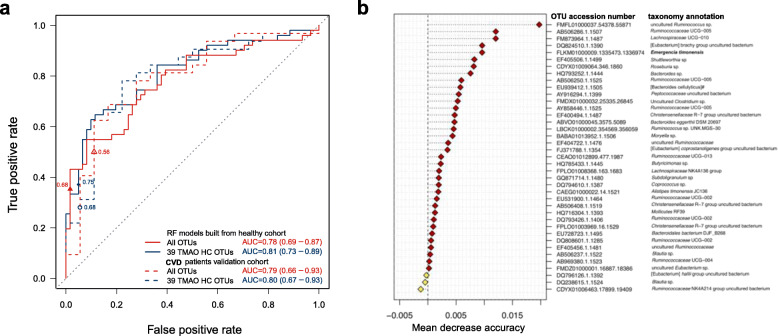
Selected TMAO-productivity-related microbiome features were used to build a TMAO producer prediction model. **a** Random forest classification modelling was built using the 39 selected OTUs, yielding a higher prediction accuracy (AUROC = 0.81) than that obtained using all 1637 OTUs (AUROC = 0.79). The prediction model was then successfully validated with an external cohort of patients with CVD (AUROC = 0.80). The 95% confidence interval of the AUCs is shown in parentheses. The optimal cutoff points were marked on the ROCs. **b** Variable importance plot of the 39 selected features ranked by mean decrease accuracy. The *E*. *timonensis* was ranked fifth among the 39 annotated taxa

Because of the distinct microbial profiles for different TMAO productivities, we then search to find signatures that could help predict TMAO producer phenotypes. To obtain the most relevant TMAO-production features, we correlated TMAO productivity with all the detected OTUs in our study cohort. We then selected the top 2.5% (empirically by the effect size of differences between microbial profiles, Fig. 4b) of the correlated OTUs as variables to establish a TMAO producer prediction model (Figure S6b). Thus, we identified 39 OTUs that were highly correlated with TMAO productivity, which also included the *E*. *timonensis* (Figure S6c and Figure S7). We then used these 39 OTUs to build a random-forest model for distinguishing high- and low-TMAO producer phenotypes and obtained a ROC curve with AUC = 0.81 (Fig. 5a). Notably, *E*. *timonensis* was ranked fifth among the 39 selected features according to mean decrease accuracy score (Fig. 5b). To confirm the prediction accuracy of the established model, we applied the model to an independent validation cohort comprising 50 patients with CVD who had received OCCT by using urine samples (Figure S8). Surprisingly, very similar prediction results with AUROC = 0.80 was established in this external validation cohort, suggesting that these microbiome features can be used to distinguish and predict human TMAO producer phenotypes from carnitine metabolism and have adequate potential to be applied in different populations. Furthermore, we compared the prediction accuracy of models developed using the 39 selected OTUs and all OTUs and found that the model developed using the selected features performed better in both the study and validation cohorts (Fig. 5a). Taken together, our results suggested that the OCCT may serve as a robust tool to identify TMAO-generating phenotypes from carnitine metabolism and to explore relevant microbiome signatures in humans.

### *Ihubacter massiliensis* is a key gut bacterium for human TMAO production

**Fig. 6:**
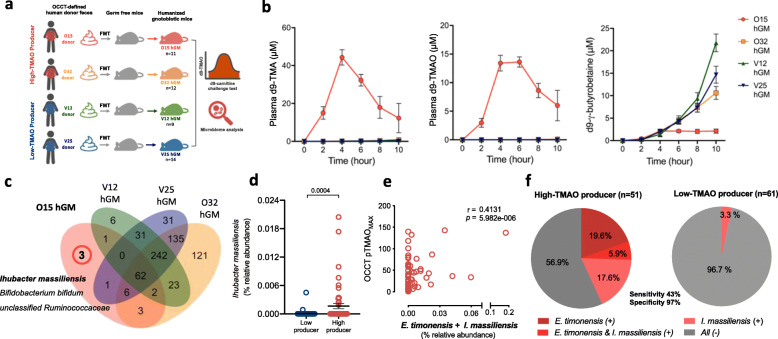
*Ihubacter massiliensis* was identified as a key bacterium for TMA/TMAO production in the humanized gnotobiotic mice (hGM) model. **a** A diagram illustrates the experiments of TMAO productivity-based hGM model. Four hGM groups were conducted through fecal microbiota transplantation (FMT) from two high-TMAO producers and two low-TMAO producers defined by oral carnitine challenge test (OCCT). **b** The d9-carnitine challenge test was performed in all groups of hGM through gavage. Only mice in the O15 hGM group demonstrated remarkable TMA/TMAO-producing ability, whereas others demonstrated undetectable d9-TMA and d9-TMAO in the plasma. The three TMA/TMAO-nonproducer hGM groups also showed increasing d9-γ-butyrobetaine (d9-γBB) level, whereas the d9-γBB in O15 hGM group was increased in the beginning and dropped latter. Time reflects hours after oral gavage of d9-carnitine. Bars represent the mean ± S.E.M for the indicated number of mice. **c** A Venn diagram showed 3 OTUs (*Ihubacter massiliensis* OTU#: LT576391.1.1479, *Bifidobacterium bifidum* OTU#: S83624.1.1532, and *unclassified Ruminococcaceae* OTU#: HQ769937.1.1427) were identified from O15 hGM group after excluding the intersections with other hGM groups. **d** *I*. *massiliensis* was significantly enriched in high-TMAO producers than low-TMAO producers defined by OCCT. Data were analyzed by paired Wilcoxon test; bars represent the mean ± S.E.M for the indicated groups. **e** The relative abundance of *E*. *timonensis* plus *I*. *massiliensis* was significantly associated with TMAO productivity (*p* < 0.0001, *r* = 0.41). **f** Among the 51 high-TMAO producers, either *E*. *timonensis* or *I*. *massiliensis* were detected in 22 subjects (43%) while *I*. *massiliensis* was detected in only 2 among 61 low-TMAO producers (3.3%). Using the presence/absence of these two bacteria in feces contributes to 43% sensitivity and 97% specificity in identifying high-TMAO producer

Although a microbiome-based prediction model may be established to predict TMAO producer phenotypes, these selected microbial features represent correlations with TMAO productivity but lack of a cause-effect relationship. Therefore, to provide a more applicable mechanistic insight, we aimed to identify the key bacteria responsible for TMAO formation from carnitine by establishing a series of TMAO-productivity-directed humanized gnotobiotic mice (hGM) models (Fig. 6a). We performed fecal microbiota transplantation to germ-free mice with feces from donors of two high-TMAO producers and two low-TMAO producers based on pre-supplement OCCT results (Figure S9a). After the colonization had stabilized, a d9-OCCT was performed with d9-carnitine via gavage to measure TMAO productivity in each hGM group. Notably, only one hGM group (O15) acquired the TMA/TMAO-producing ability from its donor, whereas the other three hGM groups had undetectable plasma d9-TMA and d9-TMAO (Fig. 6b). The O15 group also present with lowest d9-carnitine availability during the d9-OCCT (Figure S9b). Besides, the d9-γBB in the O15 hGM group increased in the beginning but dropped subsequently, whereas d9-γBB in other three hGM groups increased continuously which suggested that the d9-γBB may be consumed by the gut microbial community of O15 hGM group (Fig. 6B, right panel).

Accordingly, we suspected that the key bacteria responsible for TMA/TMAO production from carnitine may have presented specifically in the gut microbial community in the O15 hGM group and O15 donor. We first analyzed the gut microbiome profiles and found a distinguished microbial composition and diversity in each hGM group, which were relatively closer to those of their corresponding donors (Figure S9c). The difference between the hGM groups and their donors was probably attributable to the colonization preference between mice and humans. We then defined O15 hGM as the TMAO-producer group and the other three hGM groups combined as the nonproducer group. Notably, eight taxa at the genus level were significantly abundant in the TMAO-producer group (FDR < 0.01) and were found in each O15 hGM mouse (Figure S10).

Because all 11 mice in the O15 hGM group acquired TMA/TMAO-producing ability (Figure S9d), we speculated that the key TMA-producing bacteria present in the core 78 OTUs of O15 hGM mice to be acquired from the 353 OTUs of the O15 donor (Figure S9e). We then excluded the intersections between the O15 hGM and other TMAO nonproducer hGM groups, and discovered three distinct OTUs in the O15 donor/hGM group which may account for the unique TMA/TMAO-producing ability (Fig. 6c).

The three OTUs were annotated as *Ihubacter massiliensis*, *Bifidobacterium bifidum*, and an unclassified *Ruminococcaceae* by using the SILVA v132 database. According to the database, *I*. *massiliensis* is classified into the “[Eubacterium] nodatum group” at the genus level, and it was also found among the eight genera enriched in the TMAO-producer group (Figure S10). *I*. *massiliensis* is a new bacterium belonging to *Clostridiales* Family XIII that was recently isolated from human stool by the Institut Hospitalo Universitaire in Marseille, France [23]. Notably, *I*. *massiliensis* has a closest phylogenetic relationship with *E*. *timonensis* with 95% 16S rRNA sequence similarity, whereas the other 2 OTUs has distant genetic similarity to *Clostridiales* Family XIII (Figure S11a). We have also isolated *B*. *bifidum* from the sample of O15 donor and found that it does not function in converting γBB to TMA like the *E*. *timonensis* does (Figure S11b). Besides, the relative abundance of *I*. *massiliensis* was also significantly enriched in the OCCT-defined high-TMAO producer group (8.799e-5 vs. 0.002%, *p* = 0.0004) (Fig. 6d) and was positively correlated to OCCT TMAO_MAX_ (*p* = 0.02, *r* = 0.21) (Figure S12a). Furthermore, we detected a synergistic effect of *E*. *timonensis* plus *I*. *massiliensis* from their summative abundances in correlating with OCCT TMAO_MAX_ values (*p* < 0.0001, *r* = 0.41) (Fig. 6e, Figure S12b). Either the presence of *E*. *timonensis* or *I*. *massiliensis* in the feces can account for up to 43% of the high-TMAO producers, with a specificity of 97% (Fig. 6f). In addition, the presence of *E*. *timonensis* or *I*. *massiliensis* in feces contributed to an optimal diagnostic accuracy among several combinations from the selected species (Table S1) with positive predictive value of 92% for the OCCT-defined high TMAO producer. Taken together, our data suggested an extremely high likelihood that the newly found *I*. *massiliensis* is a key bacterium responsible for human TMAO production from carnitine intake, and the function may be contributed from a narrow spectrum of bacteria.

## Discussion

**Fig. 7:**
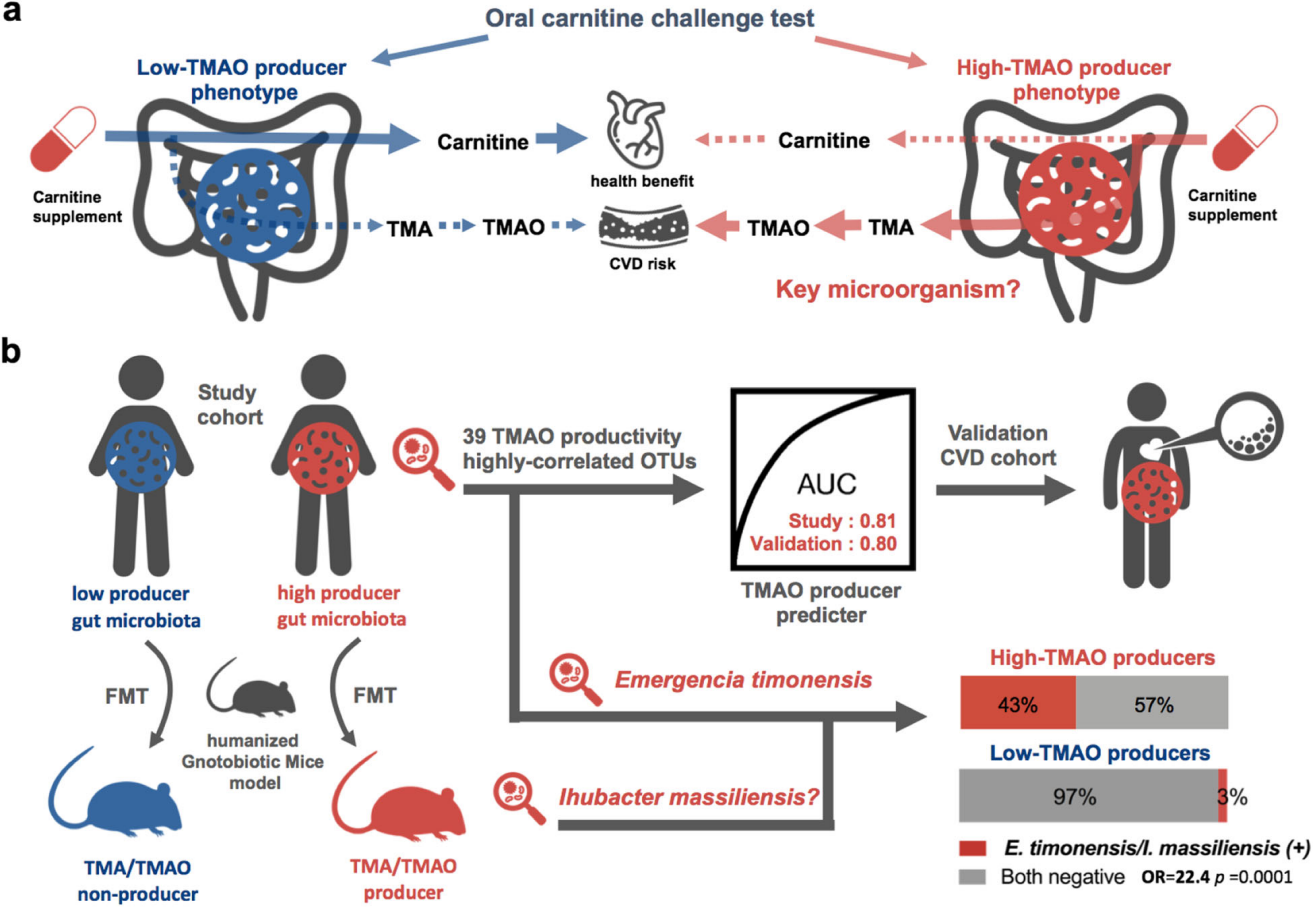
Graphical abstract. **a** The oral carnitine challenge test (OCCT) was used to classify human TMAO producer phenotypes and predict TMA/TMAO production and carnitine bioavailability from carnitine supplementation. The key gut microorganism responsible for conversion of carnitine to TMA need to be elucidated. **b** Distinct microbiome features, including *Emergencia timonensis*, were discovered between low- and high-TMAO producers; a machine learning model was created to predict the TMAO-producer phenotypes and was validated by using an external CVD patient cohort. A novel *Ihubacter massiliensis* was identified as a potential key bacterium for human TMA/TMAO production by using a humanized gnotobiotic mice model. *E*. *timonensis* and *I*. *massiliensis* together can explain 43% of high-TMAO producing status with 97% specificity

As the old saying goes, “one man’s meat is another man’s poison.” This proverb may properly describe the essence of personalized nutrition modulated by gut microbiota. Because the gut microbiota is the connecting link between diet and health, it may serve as a switch to the benefits and risks of some nutrients or drugs [1–3]. Here, we used our recently developed TMAO-producer phenotype classifier to personalize human TMAO production and carnitine bioavailability from carnitine supplementation. We discovered that TMAO productivity could be enhanced through carnitine intake as a supplement to participants’ regular diets. Moreover, we identified distinct microbiome signatures between high- and low-TMAO producers, one of which was the recently discovered *E*. *timonensis* with function of converting γBB to TMA21. Moreover, we identified 39 OTUs highly correlated to TMAO productivity from carnitine metabolism as essential features to establish a TMAO-producer prediction model, which was validated using an external cohort. Furthermore, we used humanized gnotobiotic models to identify the key TMAO-producing bacteria from carnitine and found a novel bacterium, *I*. *massiliensis*, which has a close relationship with *E*. *timonensis*. Finally, we combined the presence of either *E*. *timonensis* or *I*. *massiliensis* in the feces to predict high-TMAO producers and obtained a positive predictive value of 92%. Taken together, we demonstrated that characterization of human TMAO-producer phenotypes by using the OCCT can guide personalized carnitine intake and help identify important TMAO-relevant bacteria from the human gut (Fig. 7).

In this study, the OCCT effectively estimated a potentially harmful TMAO formation from carnitine intake; therefore, it may be used in clinical settings to reconsider whether the oral carnitine supplementation could be consumed by the microbes in generating TMA and subsequently influence the carnitine bioavailability. In our results, the low-TMAO producers demonstrated higher urine carnitine levels than high-TMAO producers after carnitine supplementation, suggesting that the TMAO-producing status could influence carnitine bioavailability. The plasma carnitine did not vary significantly which is probably because of the early absorption and rapid regulation of blood homeostasis of carnitine (within several hours). In our study, the timeline of blood sampling was designed to catch the production of plasma TMAO, therefore, may miss the optimal period for comparing plasma carnitine between groups. However, in our hGM studies, the TMAO-producing group exhibited a lower carnitine availability than other non-TMAO-producing groups during d9-carnitine challenge experiments with intensive checkpoints. Actually, a recent study also reported a negative association between serum choline and serum TMAO levels in a hGM model [24], which consists with that microbial status for TMAO production may influence carnitine/choline bioavailability. However, these important microbial effects for orally ingested nutrients and supplements have been overlooked for decades. For examples, a meta-analysis showed that carnitine supplementation in myocardial infarction patients can reduce mortality by 27% and alleviate symptoms by 40% [14]. By contrast, a more recent systematic review suggested that oral carnitine supplementation does not provide benefits in patients with CVD [15]. Importantly, all of these studies neglected the crucial metabolic effects of the gut microbiota on orally ingested carnitine, although oral carnitine supplementation had been demonstrated to have limited bioavailability and significant TMAO transformation [25–27]. Therefore, we proposed that the OCCT serves as a potential gut microbiota functional test for directing clinical decision-making and serving as a part of precision medicine. In our results, the bioavailability of oral carnitine administration may be optimal in low-TMAO producers; however, regular monitoring of plasma TMAO may be required because the phenotype of low-TMAO producer could shift to high producer in a small proportion after a 1-month carnitine supplementation.

By categorizing distinct TMAO producer phenotypes, we further aimed to identify representative microbial signatures to predict TMAO productivity. Fortunately, we identified 39 OTUs as selected features to establish a feasible random forest classifier and validated its prediction accuracy in an external cohort. Notably, we identified the *E*. *timonensis* as a high-ranking feature.

*E*. *timonensis* is a novel bacterium firstly isolated in 2016 by Bessis et al., and was recovered to convert γBB to TMA in 2019 in a pilot study conducted by Koeth et al. Since the carnitine can be converted to TMA by gut microbiota through an oxygen-independent route, with γBB as an essential intermediate, this anaerobic pathway is more likely to be responsible for carnitine metabolism in the human gut environment [21, 28]. In our study, the *E*. *timonensis* presented exclusively in the feces of OCCT-defined high-TMAO producers which provide a confirmatory evidence to the important role of this bacterium for producing TMA/TMAO from carnitine in vivo. However, in our study, the presence of *E*. *timonensis* in feces only explained 25.5% of high-TMAO producers. We thus search for additional microbial contributors of TMAO production from carnitine metabolism by using a hGM model and identified *I*. *massiliensis* as a pivotal bacterium in gut microbial TMA/TMAO production. While *I*. *massiliensis* itself accounts for 23.5% of OCCT-defined high-TMAO producers, the detection of either the presence of *I*. *massiliensis* or *E*. *timonensis* in the human gut can synergistically contribute to significant value to diagnosing high-TMAO producers, with 43% sensitivity and 97% specificity. Interestingly, *I*. *massiliensis* and *E*. *timonensis* have a closer phylogenetic relationship than other species in the clade of *Clostridiales* Family XIII. These findings may imply that the key gut microbes involved in catabolizing dietary carnitine to TMA are distributed in a relatively narrow phylogenetic spectrum. It may possibly point to an easier method for recognizing high-TMAO producers in the future through the detection of a simpler consortium or its relevant gene tag in feces (e.g., qPCR); however, additional investigations are warranted to prove the effectiveness of this approach.

Our study has some limitations. First, this is a proof-of-concept study that used a relatively small number of individuals to demonstrate microbiota-directed personalized nutrition and identify relevant microbiome features. The research findings need to be further validated in a larger cohort to confirm their generalizability in different populations. In fact, the CVD validation cohort used to validate the microbiome-based TMAO prediction model in this study had a composition distinct from that of the study cohort; however, the sample size was small. Second, the OCCT requires body fluid sampling at least three times to calculate TMAO productivity, and the inconvenience thus is caused may limit feasibility of use in clinical scenarios. Fortunately, we further proved that creatinine-adjusted urine TMAO is robust in representing plasma values with a larger sample size and may help improve the compliance of OCCT by using urine samples. Third, the intraindividual variability of OCCT at different times and the cutoff value of defining a high-TMAO producer could bring some limitation in precisely classifying high- or low-TMAO producers. For example, although we found that 7 baseline low-TMAO producers (defined by OCCT TMAO_MAX_ < 10 μM) were shifted to high-producer after carnitine supplementation, there were 6 baseline high-TMAO producers (defined by OCCT TMAO_MAX_ > 10 μM) that grouped to low producers after carnitine supplementation. The OCCT TMAO_AUC_ may exhibit a more representative TMAO-producing result than TMAO_MAX_ while its proper cutoff value remains to be determined. In our study, the test variation of OCCT does not change the fact that the low-TMAO producers were influenced more than high-TMAO producers by the carnitine supplement because the trends were consistent with several analytical results including using both data of OCCT TMAO_MAX_ and TMAO_AUC_ from plasma and urine. Finally, we speculated that *I*. *massiliensis* possesses the γBB ➔ TMA function; however, it has not been confirmed by an in vitro study despite we have tried but repeatedly failed to isolate the *I*. *massiliensis* by using published protocols, probably because of its rareness (fewer than *E*. *timonensis*). Unfortunately, it is also difficult to get access to the type strain from Marseille Institut Hospitalo Universitaire due to current COVID-19 pandemic. Although both *I*. *massiliensis* and *E*. *timonensis* are rare populations among gut microbial community with relative abundance of around 0.01% or less, however, the significance of rare species to specific biological function might still be considerable in a diverse ecological system [29]. Our results suggested an in-depth investigation may be required for some rare taxa of interest before removing them in the bioinformatic pipeline.

## Conclusion

We demonstrated the OCCT as a tool to identify gut microbial signatures and guide personalized carnitine consumption. Two obligated anaerobic bacteria, *E*. *timonensis* and *I*. *massiliensis* were identified as potential key players for converting carnitine to TMA in the human gut. We believe that these findings may facilitate precision medicine, providing future directions for mechanistic investigations and translational research.

## Materials and methods

### Oral carnitine challenge test

The OCCT was performed through oral administration of 1500 mg of carnitine tablets (GNC™) to the test participants. Blood and urine samples were collected at baseline and at 24 and 48 h after the challenge. Urine was collected within 2 h of blood sampling. An individual’s TMAO productivity was determined by calculating the AUC of the OCCT curve (OCCT TMAO_AUC_) or by using the OCCT TMAO_MAX_ level. We defined plasma OCCT TMAO_MAX_ > 10 μM (OCCT pTMAO_MAX_ > 10 μM) as the high-TMAO producer phenotype and vice versa according to a review of the literature [5, 6, 8–10]. All participants fasted overnight before the OCCT and were requested to avoid red meat, seafood, or any medication during the test. The carnitine tablets used in this study contained no animal products according to the manufacturer. More details on our creation of the OCCT can be found in a previous publication [11].

### Carnitine supplement intervention and sample collection in the healthy study cohort

A total of 56 healthy volunteers, comprising 33 omnivores and 23 vegetarians, were recruited to receive 500 mg of oral supplementation of carnitine fumarate (GNC™) daily (equals to 2–3-fold of carnitine obtained from regular diet) for 1 month. Eligibility criteria were age of 20–65 years, generally health with no chronic disease or recent illnesses, no apparent bacterial or viral infection, and no obvious gastrointestinal disorder. The participants had no exposure to antibiotics, probiotics, food supplements, or any other medication at least 1 month before the study. The OCCT was performed before and after the carnitine supplement intervention. Plasma and urine samples were collected for biochemical analysis and TMAO metabolites measurement. Each participant collected the fecal samples at home by using a validated stool collector before and after the intervention period [30]. All study protocols and informed consents of human participants were approved by the Institutional Review Board of National Taiwan University Hospital (201507055MINC), and all participants had signed a waiver of informed consent. The study for healthy subject cohort had been registered in ClinicalTrials.gov as NCT02838732. While a preliminary cross-sectional analysis of baseline data in the trial has been published, this paper presents a much more comprehensive and in-depth analysis for this interventional study.

### Measurement of Carnitine, TMAO, γ-butyrobetaine, d9-carnitine, d9-TMAO, and d9-γ-butyrobetaine

#### Sample preparation and quantification for carnitine and TMAO in healthy volunteers

Fifty microliters of plasma and 50 μL urine samples were extracted with 450 μL and 950 μL methanol containing 200 ng/mL isotopically labeled internal standards (d3-carnitine and d9-TMAO) respectively, and the extraction was performed using the Geno/Grinder 2010 (SPEX SamplePrep., Metuchen, NJ, USA) at 1000 rpm for 3 min. The extracts were then centrifuged by using the Eppendorf Centrifuge 5810R at 12,000 g for 5 min at 4 °C. The supernatants were subjected to liquid chromatography-tandem mass spectrometry (LC-MS/MS) analysis.

Target metabolites and their corresponding internal standards were analyzed by using Agilent 1290 UHPLC coupled with an Agilent 6460 triple quadrupole mass spectrometer (Agilent Technologies, Santa Clara, CA, USA). Separation was performed using a MicroSolv Cogent Diamond Hydride column (150 mm × 2.1 mm, 4.2 μm, MicroSolv, Eatontown, NJ, USA), and the column was thermostated at 40 °C during analysis. The mobile phase was composed of solvent A (10 mM ammonium acetate and 0.2% formic acid in water) and solvent B (10 mM ammonium acetate and 0.2% formic acid in 90% ACN). A 0.4 mL/min linear gradient elution was used: 0–1 min, 90–75% solvent B, 1–2 min, 75–65% solvent B, 2–4 min, 65–55% solvent B, 4–5 min, 55–40% solvent B; and column re-equilibration with 90% solvent B for 1 min. The injection volume was 5 μL. The positive electrospray ionization mode was utilized with the following parameters: 325 °C for drying gas temperature, 7 L/min for drying gas flow, 45 psi for nebulizer pressure, 325 °C for sheath gas temperature, 11 L/min for sheath gas flow rate, and 3500 V for capillary voltage. Nozzle voltage was set at 500 V. The mass spectrometer was configured in multiple reaction monitoring mode, and the monitored transitions for carnitine were *m/z* 162.1→43.2 and 162.1→60.2; d3-carnitine were *m/z* 165.1→43.1 and 165.1→61.2; TMAO were *m/z* 76.1→58.1 and 76.1→59.1; d9-TMAO were *m/z* 85.1→66.3 and 85.1→68.3. The concentration of each analyte in the samples was determined from calibration curves by using the peak area ratio of the analyte to its corresponding isotope internal standard.

#### Sample preparation and quantification for carnitine, TMAO, and γ-butyrobetaine of cardiovascular disease patient

For optimal extraction, 180 μL or 190 μL of methanol spiked with 200 ng/mL of isotopically-labeled internal standards (d3-carnitine and d9-TMAO) was added to 20 μL of plasma or 10 μL of urine samples, respectively. The samples were vortexed for 3 min and then centrifuged in Hermle Centrifuge Z216MK at 15,000 rpm for 5 min at 0 °C. The supernatants were kept for subsequent LC-MS/MS analysis.

For LC-MS/MS analysis, 20 μL of each sample was injected into Sciex Exion LC AC system coupled with SCIEX Triple TOF 5600 mass spectrometer (AB SCIEX, Canada). Separation was achieved with HILIC column (250 × 4.0 mm, 5 μm, Fortis, UK) maintained at 40 °C. The mobile phase A was 0.1% formic acid in water and mobile phase B was 0.1% formic acid in acetonitrile. The total running time was 12 min: 0–1 min, 50% solvent B, 1–9 min, 50–40% solvent B, 9–10 min, 40% solvent B, 10–10.1 min, 40–50% solvent B, followed by column re-equilibration with 50% solvent B for 1.9 min. The flow rate was 0.5 mL/min. The electrospray was set in positive ionization mode with the following parameters: 30 psi for curtain gas supply, 500 °C for the capillary temperature, 5500 V for the spray voltage floating, and 80 V for the declustering potential. The concentrations of each analyte in samples were determined from calibration curves using peak area ratio of the analyte to its corresponding isotope internal standard.

#### Sample preparation and quantification for d9-carnitine, d9-TMAO, and d9-γ-butyrobetaine of humanized gnotobiotic mice model

Twenty microliters of plasma and 10 μL of urine samples were extracted with 180 μL and 190 μL methanol containing 50 ng/mL isotopically labeled internal standards (d3-carnitine and ^13^C_3_-TMAO) respectively, and then vortex for 3 min. The extract was then centrifuged by using Hermle Centrifuge Z216MK at 15,000 rpm for 5 min at 0 °C. The supernatant was subjected to Sciex Exion LC AC system coupled with SCIEX Triple TOF 5600 mass spectrometer (AB SCIEX, Canada).

Target metabolites and their corresponding internal standards were analyzed using the same method as for the samples of cardiovascular disease patients. The concentrations of d9-carnitine and d9-TMAO in the samples were determined from the calibration curves using the peak area ratio of the analyte to its corresponding isotope internal standard. The concentration of d9-γ-butyrobetaine was calculated from the calibration curve constructed by plotting the peak area versus the concentration of d9-γ-butyrobetaine.

### 16S rRNA microbiome sequencing

Fecal samples were transported to our lab in cold storage (4–7 °C) within 24 h of collection. The feces were then aliquoted and stored at − 80 °C for microbiome analysis and other experiments. Fecal genomic DNA was extracted by using the Mobio PowerFecal DNA Isolation Kit according to the manufacturer’s instructions and quantified using the NanoDrop ND-1000 spectrophotometer (Thermo Fisher Scientific). A two-step polymerase chain reaction (PCR) workflow was conducted for library preparation in accordance with procedures described in the Illumina 16S sample preparation guide. The 16S rRNA gene V3-V4 region was amplified using a primer overhanging adapter (forward = 5′-TCGTCGGCAGCGTCAGATGTGTATAAGAGACAGC CTACGGGNGGCWGCAG-3′ and reverse = 5′-GTCTCGTGGGCTCGGAGATGTGTATAAGAG ACAGGACTACHVGGGTATCTAATCC-3′). Dual indices and Illumina sequencing adapters were attached through PCR by using a Nextera XT Index Kit according to the manufacturer’s instructions. After each PCR process, PCR cleanup was performed using AMPure XP beads to purify V3–V4 amplicon from the free primer and primer dimer. The sizes of PCR products were verified using the Bioanalyzer DNA 1000 chip. Library quantification was performed for quality control before sequencing by using the Agilent Technologies 2100 Bioanalyzer. The pooled libraries were then sequenced on the Illumina MiSeq platform with v3 reagents for paired-end sequencing (2 × 300 bps).

### Bioinformatics analysis

#### 16S-Amplicon processing pipeline

The 16S-amplicon processing pipeline was modified from 16S Bacteria/Archaea SOP v1 of Microbiome Helper workflows [31]. Paired-end reads were assembled by using PEAR v0.9.8 [32]. Assembled sequences were quality-filtered by thresholds of sequence length ≥ 400 bp and quality score of 90% bases of reads ≥ 20. All quality-filtered reads were analyzed with the pipeline of QIIME (v1.9.1) pipeline [33, 34]. OTUs were assigned using a closed-reference OTU picking approach, which referenced picks against SILVA (NR132) database [35, 36] by using UCLUST algorithm [37] with 97% of sequence identity. The generated OTU table was filtered by removing singletons as well as low-confidence OTUs which, which underwent bleed-through removal between MiSeq runs. The final OTU table was rarefied into 61,600 reads/sample.

#### Mining low-abundance sequences by using the BLAST algorithm

*I*. *massiliensis* failed to pass the confidence-filtering of the OTU table because of its low read abundance in the sequence library. We retrieved the 16S rRNA gene sequence of *I*. *massiliensis* strain Marseille-P2843 from NCBI GenBank database (accession number NR_144749.1) [23]. The absolute read counts of *I*. *massiliensis* were then extracted from quality-filtered reads by using *blastn* algorithm [38] (*e* value < 1e-5, identity ≥ 97%) by searching against the 16S rRNA gene sequence of *I*. *massiliensis*.

### Biodiversity and statistical analyses

Gut microbial community analyses were conducted with the R package *vegan* [39]. A Mann-Whitney *U* test in R software [40], with *α* = 0.05, was used for statistical analyses. Multiple-testing *P* values were adjusted with FDR by using “p.adjust” function in R software. Alpha diversity indices and Shannon index were calculated by “diversity” function; observed OTU was counted by “specnumber” function; and the Chao1 index was calculated by “chao1” function of *fossil* package [41]. For beta diversity, dissimilarities among microbial communities were measured by Bray-Curtis distance and principal coordinates analysis; ADONIS (permutational multivariate analysis of variance using distance matrices) was used to test the heterogeneity of gut microbial composition among sample groups [42]. The associations between TMAO-producing phenotypes (Log10 urine TMAOMAX) and gut microbial abundance were measured using Pearson’s correlation coefficients by using log10-transformed OTU abundances. The top 2.5% highly correlated OTUs (39 of the 1637 OTUs) were selected for subsequent phylogenetic analysis and random forest modelling. To profile the OTU abundance alterations among samples/groups or across TMAO concentrations, a heatmap was generated by using *pheatmap* R package [43]. The statistical analysis for comparing serum biochemical values and TMAO levels were evaluated by Student’s *t* test, Wilcoxon rank-sum test, and Mann-Whitney *U* test accordingly and appropriately using Prism 8.0 software based on data distribution and characteristics.

### Phylogenetic analysis

Phylogenetic analysis of associated TMAO-producing OTUs was conducted using an online phylogeny analysis pipeline (Phylogeny.fr) [44, 45]. The “One Click” mode was selected for the following analyses: the candidate 16S rRNA gene sequences were first submitted in the fasta format; sequences were aligned with MUSCLE [46], and aligned sequences were curated with Gblocks [47]; the phylogenetic tree was constructed with PhyML by using the maximum likelihood method [48]; a constructed phylogenetic tree was visualized and rendered by FigTree v1.4.3 [49].

### Cardiovascular disease patient validation cohort

A total of 50 patients with catheterization-proven CVD (> 50% stenosis for at least one coronary vessel) were recruited as an independent cohort for validating the microbiome-based TMAO producer prediction model (the baseline characteristics of CVD validation cohort were described in Table S2). The patients with CVD received a simplified OCCT by urine sampling and were categorized according to urine OCCT TMAO_MAX_ level (uOCCT TMAO_MAX_). Because the urine and plasma TMAO levels were highly correlated, a cutoff value of OCCT uTMAO_MAX_ ≥ 162.79 mmol/mol creatinine, corresponding to OCCT pTMAO_MAX_ > 10 μM, was defined for high-TMAO producers on the basis of the equation of the linear regression plot between urine and plasma TMAO levels (Supplementary Figure 6A). Stool samples were collected using the same process as that described for microbiome analysis in the healthy study cohort. The study protocol and informed consent obtained from patients with CVD were approved by the Institutional Review Board of National Taiwan University Hospital (201712030RIND) and all participants in this study had signed a waiver of informed consent. The study for CVD patient cohort had been registered in ClinicalTrials.gov as NCT03781011.

### Random forest classification modelling for TMAO producer phenotypes prediction

Random forest classification models were built for predicting high- or low-TMAO producing phenotypes. A healthy cohort (112 samples from 56 subjects receiving OCCT for two times) was used for building predication models with *randomForest* R package [50]. An external CVD cohort with 50 participants served as a validation cohort for the prediction model. Two prediction models were built, one with 39 high-TMAO-correlated OTUs and the other with the whole OTU dataset (1637 OTUs). Leave-one-out cross-validation was performed for estimating model accuracy by using *caret* R package [51]. The importance of features was then ranked using the values of “mean decrease accuracy.” Finally, the model accuracy was measured according to the AUROC by using the *ROCR* package in R software [52].

### Humanized gnotobiotic mouse experiments

C57BL/6JNarl male germ-free mice (8–12 weeks of age) were used to establish four study groups of hGM model through fecal microbiota transplantation (FMT) from donors of two OCCT-defined high-TMAO producers (O15 and O32) and two OCCT-defined low-TMAO producers (V12 and V25). Each hGM group was established by gavaging germ-free mice with 200 μL of human fecal suspension, which was prepared by using a 1 g aliquot of feces diluted in 10 mL of reduced phosphate-buffered saline (supplemented with 0.05% l-cysteine) in an anaerobic chamber. After the colonization had stabilized (at least 2 weeks after FMT), the hGM received oral carnitine challenge test by gavage administration with d9-carnitine (150 μL from a 150-mM stock) dissolved in water. Plasma was collected from the submandibular vein at baseline and at indicated times.

The hGM experiments were performed using the facilities of the National Laboratory Animal Center, National Applied Research Laboratories, Taiwan, and were approved by the Institutional Animal Care and Use Committee (IACUC2017O04). Their germ-free mice maintenance protocol is as follows: mice are maintained in a vinyl isolator (CBC, Madison, WI) at the room the temperature of 22 ± 1 °C and 55–65% relative humidity with a 12-h light/12-h dark cycle. Mice are fed a commercial diet (5010 LabDiet, Purina Mills, St. Louis, MO) and sterile water ad libitum. To confirm GF status, microbiological assays were performed on a monthly basis by culturing feces, bedding, and drinking water in thioglycollate medium (DIFCO, Camarillo, CA).

## Supplementary information

**Additional file 1:.** Tables S1-S2 and Figures S1-S12

## Data Availability

The sequence read data of gut microbiome for characterizing TMAO producers (BioProject:PRJNA589723) were all available on NCBI sequence read archive (SRA) database (SRA accession numbers:SRR10465787-SRR10466048).
