# Supplementary material for: Characterization of TMAO productivity from carnitine challenge facilitates personalized nutrition and microbiome signatures discovery
Source: Microbiome. 2020 Nov 19;8:162. doi: 10.1186/s40168-020-00912-y (PMC7676756; doi:10.1186/s40168-020-00912-y)
Supplement: Supplementary file 2 — Additional file 1:. Tables S1-S2 and Figures S1-S12 [file 40168_2020_912_MOESM1_ESM.docx]

**Additional files**

**Table S1. Diagnostic accuracy of the presence of selected bacteria for detecting high-TMAO producers**

|  | Sensitivity | Specificity | PPV | NPV | Diagnostic accuracy |
| --- | --- | --- | --- | --- | --- |
| *E. timonensis* (+) | 31.4% (16/51) | 100% (61/61) | 100% (16/16) | 63.5% (61/96) | 68.8% (77/112) |
| *I. massiliensis* (+) | 23.5% (12/51) | 96.7% (59/61) | 85.7% (12/14) | 60.2% (59/98) | 63.4% (71/112) |
| *B. bifidum* (+) | 70.6% (36/51) | 57.4% (35/61) | 58.1% (36/62) | 70% (35/50) | 63.4% (71/112) |
| *E* (+) or *I* (+) | **47.1% (24/51)** | **96.7% (59/61)** | **92.3% (24/26)** | **68.6% (59/86)** | **74.1% (83/112)** |
| *E* (+) or *B* (+) | 80.4% (41/51) | 57.3% (35/61) | 61.2% (41/67) | 77.8% (35/45) | 67.9% (76/112) |
| *E* (+) or *I* (+) or *B* (+) | 80.4% (41/51) | 55.7% (34/61) | 60.3% (41/68) | 77.3% (34/44) | 67.0% (75/112) |

PPV, positive predictive value; NPV, negative predictive value

**Table S2. Baseline characteristics of CVD validation cohort (n = 50)**

| *Demographic* |  | *Biochemistry* |  | *Medication* |  |
| --- | --- | --- | --- | --- | --- |
| Gender | Male 92% | Glucose-AC (mg/dL) | 95.3 ± 4.22 | Antiplatelet | 100% |
| Age | 55.18 ± 6.46 | HbA1c (%) | 6.0 ± 0.12 | Statin | 80% |
| BMI (kg m^-2^) | 22.40± 0.55 | AST (U/L) | 23.9 ± 0.88 | Metformin | 22% |
| Hypertension | 84% | ALT (U/L) | 29.76 ± 2.25 |  |  |
| Hyperlipidemia | 84% | Creatinine (mg/dL) | 1.02 ± 0.05 |  |  |
| Diabetes mellitus | 34% | T-Cholesterol (mg/dL) | 148.84 ± 4.26 |  |  |
| CAD status | 1VD 28%  2VD 20%  3VD 52% | Triglyceride (mg/dL) | 136.78 ± 11.46 |  |  |
| OCCT high-TMAO producer | 66% | HDL-C (mg/dL) | 43.84 ± 1.50 |  |  |
|  |  | LDL-C (mg/dL) | 90.22 ± 3.62 |  |  |
|  |  | CRP (mg/dL) | 0.16 ± 0.02 |  |  |
|  |  | TMAO (µM) | 7.74 ± 1.35 |  |  |

1VD: one coronary vessel disease; 2VD: one coronary vessels disease; 3VD: one coronary vessels disease diagnosed by coronary angiography

**Figure S1. Homeostasis of TMAO and carnitine are regulated by renal system in different ways. a** The fasting urine carnitine was increased significantly after carnitine supplementation while carnitine in plasma showed no significant change. Both fasting plasma and urine TMAO showed significant increases after carnitine supplementation. **b** The plasma and urine TMAO levels were highly (Pearson r = 0.83-0.90) correlated for both logarithmic and original data (*p* < 0.0001). **c** The plasma and urine carnitine levels were moderately (Pearson r = 0.20-0.36) correlated (*p* = 0.0003). The scatter plot at right column also showed a narrowed distribution of plasma carnitine between 20-60 μM while urine carnitine showed nonparametric distribution. **d** The fractional excretion of carnitine (FeCarnitine) showed 0.36% (IQR = 0.15-1.00%) to 0.93% (IQR = 0.45-2.21%) while the fractional excretion of TMAO (FeTMAO) showed 83.6 (IQR = 66.5-108%) to 93.85% (IQR = 67.7-110%). The FeCarnitine showed a significant increase after carnitine supplementation. **e** Schematic diagram showing renal regulation of TMAO and carnitine homeostasis in the human circulation reservoir pool. These data suggest the kidney tends to eliminate TMAO as much as possible while to reserve carnitine not less than 20μM in the blood.

**Figure S2. TMAO productivity in low TMAO producer was enhanced by carnitine supplementation regardless of omnivorous or vegetarian diets. a** The OCCT measured with urine TMAO showed consistent results with plasma samples. The TMAO productivity measured by urine samples in low-TMAO producer was significantly increased after carnitine supplementation, while it showed no significant change in high-TMAO producer. **b** The TMAO productivity measured by using urine TMAO_MAX_ also showed consistent results. **c** and **d** Data sub-grouped by dietary habits continued to show significant increases of TMAO productivity in low-TMAO producers in both omnivorous and vegetarian groups after carnitine supplementation, but no significant change in high TMAO producer.

**Figure S3. a** No significant change of microbial profiles was noted after carnitine supplementation for either vegetarian or omnivorous groups by using principle coordinate analysis with Bray-Curtis distance. **b** No significant change of microbial profiles was noted after carnitine supplementation for either low- or high-TMAO producers by using principle coordinate analysis with Bray-Curtis distance.

**Figure S4. a** It also showed no significant difference of microbial profiles between samples regrouped according to the fasting plasma TMAO level with a cut-off value of 6.2 μM. **b** For the 51 samples corresponding to high-TMAO production status (TMAO_MAX_ >10 μM), only 21.6% were noted to have fasting plasma TMAO greater than 6.2 μM. **c** Most samples (84.6%) with fasting plasma TMAO > 6.2 μM had TMAO_MAX_ values greater than 10μM in OCCT, however, a significant portion (40.4%) with fasting plasma TMAO < 6.2 μM were grouped as high-TMAO producer (TMAO_MAX_ >10 μM) by OCCT. **d** The relative abundance of *E. timonensis* between fecal samples with fasting plasma TMAO < 6.2 μM vs fasting plasma TMAO > 6.2 μM was not significantly different.

**Figure S5.** 101 OTUs including the *Emergencia timonensis* from a total 1637 OTUs were identified significantly enriched in TMAO high producers than low producers (FDR <0.01) and was demonstrated in a heatmap.

**Figure S6. Top 2.5% OCCT TMAO_MAX_ highly-correlated 39 OTUs were selected as features to build a predictive machine learning model. a** A urine cut-off value of 162.79 mmol/mol creatinine corresponding to plasma OCCT TMAO_MAX_ = 10 μM was obtained by using linear regression curve for defining high- and low-TMAO producer in both study and validation groups. **b** The summative relative abundance of the top 2.5% TMAO-productivity-correlated OTUs (39 selected OTUs) in the 112 fecal samples was significantly correlated to TMAO productivity, with Pearson’s r = 0.56 (*p* <0.001). **c** A phylogenetic tree constructed using the 39 selected OTUs showed a major phylum clade of *Firmicutes* with subdivisions of *Clostridiales* Family XIII, *Peptococcaceae*, *Ruminococcaceae*, *Christensenellaceae*, and *Lachnospiraceae* at the family level. *Emergencia timonensis* was detected within the 39 selected OTUs and belongs to *Clostridiales* Family XIII. *Emergencia timonensis* SN18 and an *Escherichia coli* were placed on the tree as referenced species.

**Figure S7.** Top 2.5% OTUs highly correlated to TMAO productivity (39 OTUs, including *Emergencia timonensis*) in the health subjects were demonstrated with heatmap and selected as microbiome features for TMAO producer phenotype classifier.

**Figure S8.** A heatmap demonstrates the selected 39 OTUs in the CVD validation cohort with its high-TMAO producer defined by OCCT uTMAO_MAX_ > 162.79 mmol/mol creatinine.

**Figure S9. a** The OCCT diagram of selected human donors for establishing a humanized gnotobiotic mice model by performing FMT to germ-free mice. **b** The O15 hGM group demonstrated the lowest carnitine availability from oral gavage of d9-carnitine as compared with other hGM groups (data presented as percentage compared with the mean concentration at 2^nd^ hour). **c** The alpha diversity of each hGM group were generally lower and similar to that of its donor. The horizontal lines indicate the alpha diversity value of donors; box plots showed a median with 25^th^ and 75^th^ percentiles and whiskers denote min to max value. **d** The eleven O15 hGM mice all acquired the TMA/TMAO producing ability by fecal microbiota transplantation from the O15 donor. **e** The principle coordinate analysis plot revealed distinct microbial profiles for mice in each hGM group. The microbial composition for each hGM group was relatively close to its donor. 78 core OTUs in O15 hGM are acquired from the O15 donor.

**Figure S10.** Eight taxa at the genus level, including the [Eubacterium] nodatum group, had a significantly higher abundance in the TMAO-producer hGM group than in all other TMAO-nonproducer hGM groups (FDR <0.01). We further identified the OTU belonging to [Eubacterium] nodatum group in this hGM study as *Ihubacter massiliensis.*

**Figure S11.** *Ihubacter massiliensis* and *Emergencia timonensis* have the closest phylogenetic relationship. **a** A phylogenetic tree exhibited the relationship of *Ihubacter massiliensis* (OTU#: LT576391.1.1479), *Bifidobacterium bifidum* (OTU#: S83624.1.1532), and the uncultured *Ruminococcaceae* (OTU#: HQ769937.1.1427) to bacteria in the *Clostridiales* Family XIII revealed that *Ihubacter massiliensis* and *Emergencia timonensis* have the closest relationships, with a 95% 16S rRNA gene sequence similarity. **b** The *in vitro* study incubated with d9-γ-butyrobetaine (d9-γBB) showed *E. timonensis* was capable of converting γBB to TMA while *B. bifidum* was not.

**Figure S12. a** The relative abundances of OTUs assigned to *I. massiliensis* and *B. bifidum* were significantly correlated to the TMAO productivity. **b** The summative abundances of *E. timonensis* and *I. massiliensis* exhibited a synergistic correlation to TMAO productivity.
